# Improved Documentation of Electronic Cigarette Use in an Electronic Health Record

**DOI:** 10.3390/ijerph17165908

**Published:** 2020-08-14

**Authors:** Thulasee Jose, J Taylor Hays, David O. Warner

**Affiliations:** 1 Department of Anesthesiology and Perioperative Medicine, Mayo Clinic, Rochester, MN 55902, USA; warner.david@mayo.edu; 2 Department of Medicine, Mayo Clinic, Rochester, MN 55902, USA; hays.taylor@mayo.edu

**Keywords:** e-cigarettes, vaping, electronic nicotine delivery device, electronic cigarettes

## Abstract

The use of electronic cigarettes (e-cigarettes) can affect patient health and clinical care. However, the current documentation of e-cigarette use in the electronic health records (EHR) is inconsistent. This report outlines how the ambulatory clinical practices of a large U.S. hospital system optimized its electronic health records (EHR) framework to better record e-cigarettes used by patients. The new EHR section for e-cigarette information was implemented for outpatient appointments. During a 30-week evaluation period post-implementation, 638,804 patients (12 yrs and older) completed ambulatory appointments within the health system; of these, the new section contained e-cigarette use information for 37,906 (6%) patients. Among these patients, 1005 (2.7%) were identified as current e-cigarette users (current every day or current some day e-cigarette use), 941 (2.5%) were reported as former e-cigarette users, and 35,960 (94%) had never used e-cigarettes. A separate EHR section to document e-cigarette use is feasible within existing clinical practice models. Utilization of the new section was modest in routine clinical practice, indicating the need for more intensive implementation strategies that emphasize the health effects of e-cigarette use, and how consistent ascertainment could improve clinical practice.

## 1. Introduction

Cigarette smoking is the leading cause of preventable disease and death in the United States [1]. Despite the overall progress made to curb combustible cigarette smoking in the U.S. (the prevalence of smoking was 13.7% in 2018), between 2014 and 2018, the prevalence of electronic cigarette (e-cigarette) use increased from 5.1% to 7.6% in U.S. adults aged 19–24 years [2]. E-cigarettes are battery-powered devices that deliver an aerosol by heating solutions that usually contain nicotine, propylene glycol, and a variety of other solvents and flavoring agents [3]. The devices (also known as “vapes”), can be used to deliver other substances, including tetrahydrocannabinol (THC) [4].

According to the World Health Organization (WHO), many countries lack tobacco surveillance and monitoring systems to effectively assess the prevalence of current e-cigarette use [5]. A recent report suggested that in mid-2018, at least 40 million adults across the globe used e-cigarettes [6]. Although these devices may function as nicotine delivery systems to help adult cigarette smokers quit, they may also serve as a means to initiate or perpetuate nicotine addiction in adults and the youth. In addition, the potential dangers and health consequences of e-cigarette use were illustrated by the outbreak of e-cigarette, or vaping, associated lung injury (EVALI) [7,8]. Although many patients who developed EVALI used products containing tetrahydrocannabinol, some used devices containing only nicotine [9].

For these reasons, it is important for clinicians to accurately ascertain and record e-cigarette use in their patients. However, the current documentation of e-cigarette use in the electronic health records (EHR) is inconsistent [10]. The evidence suggests that many clinicians record e-cigarette information within the “tobacco use” section of the EHR, along with multiple combustible tobacco products (cigarettes, cigars, pipes, etc.), or as free-text entries as a part of their clinical notes [11,12]. These documentation efforts are not consistent across clinical practices, nor systematically tracked by the health system. Reliable population health data could bolster research efforts to generate evidence addressing gaps in our understanding of trends in e-cigarette use by patients and their overall impact on health [10,13,14]. These data are also important for clinicians to deliver optimal care, as many may not even be aware of their patients’ e-cigarette use [15].

The current version of the EHR system used by the health system does not utilize a separate section to document e-cigarette use, but rather employs a single tab called “E-cigarettes” within the “Tobacco Use” section of the social history, that clinicians can use to indicate every use of e-cigarettes. However, it is not possible with this tab to separately describe cigarette and e-cigarette use for dual users. The anecdotal experience suggested that clinicians were not consistently capturing e-cigarette use (perhaps because many clinicians do not perceive e-cigarettes to be a tobacco product). Therefore, our objective was to design and institute a separate documentation framework within the EHR system to capture e-cigarette use information from patients, and to record this information separately from other tobacco product use information. This report describes the design and pilot implementation of this documentation system within a large U.S. hospital system.

## 2. Materials and Methods

**Clinical Setting:** Mayo Clinic is a large academic U.S. hospital system, with over 4000 physicians serving approximately 1.5 million patients annually, with primary campuses in Minnesota, Florida, and Arizona. The clinical practice is supported by an EHR vendor, Epic© Systems Corporations. The design and implementation work described in this report was performed at the Mayo Clinic in Rochester, MN and applicable to all ambulatory practices within the health system.

**Prior EHR documentation of e-cigarette use:** Prior EHR documentation of e-cigarette use was limited to single tab within a “tobacco use” section of the “substance and sexual activity” history. Note that for users of more than one tobacco product, it is not possible to ascertain status (i.e., current user, former user, etc.) for each product. For example, for dual users of cigarettes and e-cigarettes, it is not possible to determine whether they are currently using both, or have discontinued the use of one. Indeed, clinicians and patients may not consider that e-cigarette use falls under “smoking status”, as their use may not be considered to be “smoking”. In addition, tobacco use information within the substance and sexual activity history can be updated by a clinician at any time, and need not be within the context of an appointment with the patient. Typically, this section is updated as part of a clinical encounter with the patient. However, in the event that patients are not asked about their tobacco use history during a clinical appointment, information that is currently on file would remain as is. The current institutional policy for ambulatory clinical practices mandates that tobacco use history must be ascertained and updated, at least once a year for all patients age 12 and over, although individual practices may update more frequently. Our preliminary request to Epic© was let us optimize the “tobacco use” history, and include a separate section for e-cigarette information. This request was initially declined, citing that the tobacco-related documentations entered in this section were being used for regulatory requirement reporting purposes. As a result, we proposed to design and pilot the new e-cigarette documentation framework for the ambulatory clinical practices and utilize this as a template for Epic©’s future optimization efforts.

1. **New e-cigarette documentation framework design:** The new EHR documentation framework was designed for the outpatient clinical practices of the health system. The design considerations for the new framework included the following:
2. Provide a distinct section within the social determinants of the health section for e-cigarette use documentation, separate from the “tobacco use” information within the substance and sexual history section;
3. Provide the ability to record the frequency of e-cigarette use, types of devices, the types of substances used, the start date, the end date, counseling status; and
4. Provide the option for free entry of information by clinicians, given the wide range of available devices and substances that could be used.

The new e-cigarette section is designed to be available only during ambulatory appointments and must be completed by a clinical provider (not the patient) as part of the routine patient health history. Unlike the previous documentation system where information on the patient file could be updated by a clinician at any time, the new section to record e-cigarette information is made available only within the context of a “clinical encounter with the patient” and cannot be filled outside the “ambulatory appointment”. This feature was designed to encourage providers to complete this section as part of the patient visit. Per current institutional policy, information collected in this section is not a “required” component of the EHR and, therefore, clinicians are allowed to skip the new section. Examples of “required” components of an EHR include patient name, age, ethnicity, gender, address, insurance information, etc. Only a highly selective group of information is considered as a “mandatory” requirement for all patient EHRs. The remaining sections, including the patient medical history sections, are filled out based on institutional, departmental, and clinical practice guidelines. This is done intentionally to ensure patient EHRs can be created to provide medical services during urgent situations without delay.

The development, testing, and implementation of the new documentation framework were conducted at the Rochester campus, with additional oversight from Epic©. The content, layout, and the location of the new section were finalized after consulting with tobacco treatment specialists at the Mayo Clinic Nicotine Dependence Center and the evidence based guidelines in this domain [16,17]. It was approved for use by institutional clinical practice and EHR oversight committees.

**Implementation:** Prior to the implementation of the new framework, an online newsletter communication was delivered to all clinicians, announcing the importance of using the new section as part of ambulatory clinical practice. On the week of implementation, the new upgrade was announced on the EHR update webpage run by the hospital system. No other specific implementation activities were conducted.

**Analysis:** The utilization rate of the new section in EHR was assessed by a report of the total number of responses recorded for the selected variables (i.e., e-cigarette use, types of devices, and counseling status) during the evaluation period. The 30 weeks between 26 February 2019–21 September 2019, was considered as the pre-implementation period. The new section was implemented in the EHR system on 27 September 2019. A one-week period between 27 September and 6 October 2019, was considered as a “run-in” period and was not used for evaluation. The evaluation period for the new e-cigarette section was 30 weeks, from 7 October 2019, to 30 April 2020.

## 3. Results

**Figure 1:**
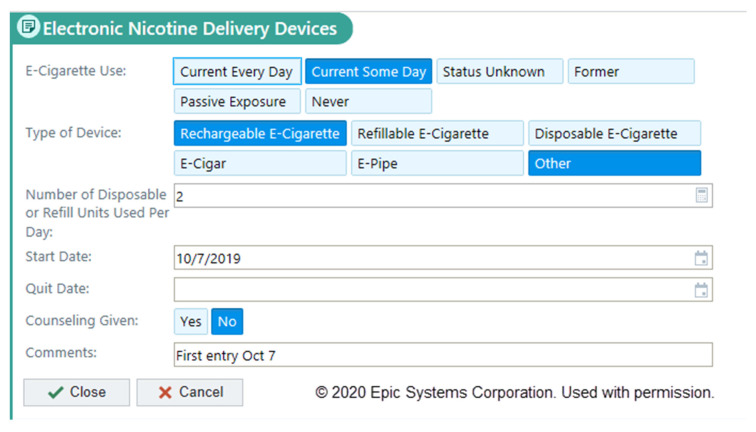
New electronic health record documentation framework to record the e-cigarette use information collected from patients during ambulatory appointments.

The new section on e-cigarettes is the first tab under the social determinants of the health section of the EHR and is labeled as “Electronic Nicotine Delivery Devices” (Figure 1). This descriptor was selected to distinguish from the traditional combustible nicotine delivery devices (i.e., cigarettes, cigars, cigarillos, hookah, etc.) and chewing tobacco types that are recorded under the tobacco use section. In the newly designed section, discrete choices are provided to document the frequency of e-cigarette use, the type of device used, the number of disposable or refill units used per day, the date when e-cigarette use began, the date when the patient quit using e-cigarettes (for former users), if the provider performed counseling for the patient about e-cigarette use, and an open text box to write additional information provided by patients (i.e., brand names of e-cigarettes, user behaviors associated with vaping, etc.).

The new e-cigarette section was implemented across the ambulatory clinical practices of the health system on 27 September 2019. During the 30-week evaluation period subsequent to implementation, a total of 1914,353 outpatient visits were completed by 638,804 patients (age ≥ 12 years old) across the health system. Of these, the records of 37,906 (6%) patients had information in the new e-cigarette section completed by a clinician. A summary of total responses recorded in the sections is presented in Table 1.

Of the 37,906 patients with a completed “frequency of use” section, 23,221 (61%) were female. Regarding age, 1782 (5%) were less than 18 years old, 3293 (9%) were between 18-25 years old, 10,293 (27%) were between 26–45 years old, 12,151 (32%) were between 46–65 years old, and 10,387 (27%) were more than 65 years old.

A total of 666 (1.8%) patients reported current every day use, 339 (0.9%) patients reported current some day use and 941 (2.5%) reported former use of e-cigarettes. The most commonly used type of e-cigarette device was refillable, followed by rechargeable devices and disposable types of devices. The counseling status section was completed for 4391 patient records; 776 (17.6%) patient records indicated counseling on e-cigarette use by clinicians. Counseling was provided by clinicians to 355 of 1005 (35.3%) current or some day e-cigarette users, 91 of 941 (9.6%) former e-cigarette users, and 321 of 35,777 (0.9%) never users.

## 4. Discussion

There is a growing need for more training, research, and support for clinicians regarding e-cigarette use among patients, given their significant impacts on health [15,18,19]. The overall prevalence of reported current e-cigarette use among U.S. adults was reported as 3.2% in 2018, including 7.6% of those 18 to 24 years-old [20]. This overall rate of use is consistent with the rates observed in those patients with information entered in the new section, with 2.6% of patients reporting every day or some day use, and 2.5% reporting former use. Prior reports suggest that e-cigarette use information appears to be under-reported in the EHR [11,12,14]. Studies examining e-cigarette use information in the EHR suggest several deficiencies, including misclassification and inconsistent documentation practices [10,11,14], with the general lack of structured fields in EHRs to document e-cigarette use serving as one potential barrier [21]. On the other hand, by increasing the already considerable provider, the EHR burden could become counterproductive if systems are not carefully designed. The EHR systems undergo frequent upgrades, and clinicians need to quickly adapt to such changes [22]. Designing systems to record e-cigarette use is particularly challenging because clinicians may not routinely screen patients for e-cigarette use [23].

The new e-cigarette section is distinct from the documentation of other tobacco products. Reasons for this separation include (1) some e-cigarette products do not contain nicotine; (2) clinicians may not realize that e-cigarettes are tobacco products, and (3) a separate section may also serve as a cue for clinicians to systematically record e-cigarette use. Previously, clinicians had to record any additional information about e-cigarettes within clinical notes. The new framework still allows limited free text entry by clinicians to enter information as reported by patients. For example, a free text entry from a clinician read “Quit when she found out she was pregnant and when coronavirus concerns occurred”, suggesting that clinicians can find this feature useful. However, the use of structured data fields facilitates the conduct of digital health surveillance of patients that use these devices, and provides the opportunity to generate clinical and analytical reports that can support research investigations examining the epidemiology, potential risks, and impact of e-cigarette use.

The utilization of the new section by the clinical staff was modest (with information entered for only 6% of patients). Several factors could have contributed. Our implementation effort in terms of educating clinicians was minimal (i.e., a one-time online communication newsletter sent to providers), so many clinicians may not be aware of the new section. The added documentation also increases the clinician burden. Although we intended to improve the capture of e-cigarette use information, we transitioned from a single checkbox documentation system to a separate section with six new questions and space for free text entry. Adding more documentation fields could impact the clinical workflow and increase the overall EHR documentation burden [24]. Possible solutions would be to allow self-entry of medical history by the patients (e.g., allow patients to complete own medical history via online patient portal prior to outpatient appointments), assigning other members of the care team (i.e., patient scheduling team, medical assistants, or rooming staff) to record medical history, and engaging the clinical practice leadership to advocate for systematic ascertainment of e-cigarette use. Finally, clinicians may be less likely to complete this section if patients do have a history of e-cigarette use, although the finding that the use rates noted among patients with this section completed are consistent with national data argues against this possibility. Clearly additional implementation efforts are indicated to increase the utilization of this new section, and planning for these efforts is underway.

One limitation of our work is that we were not able to compare clinician utilization of the new section with the section used in prior documentation (i.e., the single tab in the “tobacco use” section), due to the different procedures used to update each section. Future evaluation studies would be necessary to compare different methods of recording e-cigarette use.

## 5. Conclusions

A separate EHR section to document e-cigarette use is feasible and provides a consistent approach to assess e-cigarette use in both youth and adult populations. This implementation was the first of its kind for Epic©, one of the largest EHR vendors in the U.S. This work ultimately contributed to the 2020 Epic© foundation build upgrade that optimized the tobacco use history, including a similar separate section to record e-cigarette use information. Utilization of the new section was modest in routine clinical practice, indicating the need for more intensive implementation strategies that emphasize the health effects of e-cigarette use, and how consistent ascertainment could improve clinical practice.

## Figures and Tables

**Table 1:** E-cigarette information recorded by clinicians between 7 October 2019, to 30 April 2020. The number represents cumulative data during the time period.

Categorical Choice	Response Choices	Number of Patients
Electronic Cigarette Use:	Current Every Day	666
	Current Some Day	339
	Former	941
	Passive Exposure	55
	Never	35,777
	Status Unknown	128
Type of device:	Rechargeable E-Cigarette	392
	Refillable E-Cigarette	507
	Disposable E-Cigarette	89
	E-Cigar	28
	E-Pipe	4
	Other	58
Cessation Counseling Given	Yes	776
	No	3615
